# Association of feeding methods in early life with gut microbiota in neonates

**DOI:** 10.3389/fmicb.2026.1921787

**Published:** 2026-09-11

**Authors:** Huimin Lai, Xiongfeng Pan, Shiting Xiang, Yunlong Peng, Kunyan Zhao, Sha Wu, Xiaoming Peng, Yiying Kuang, Cai Gao, Jun Qiu, Songxu Peng

**Affiliations:** 1 Department of Maternal and Child Health, Xiangya School of Public Health, Central South University, Changsha, Hunan, China; 2 Pediatrics Research Institute of Hunan Province, Hunan Children’s Hospital, The Affiliated Children’s Hospital of Xiangya School of Medicine, Central South University, Changsha, Hunan, China; 3 Department of Epidemiology and Health Statistics, Medical College of Soochow University, Suzhou, China; 4 The School of Public Health, University of South China, Hengyang, China; 5 Department of Pediatrics, Hengyang Medical School, University of South China, Hengyang, China; 6 Department of Neonatology, Hunan Children's Hospital, Changsha, Hunan, China

**Keywords:** early life, feeding methods, gut microbiota, neonates, preterm neonates

## Abstract

**Background:**

The research on the gut microbiota of neonates and preterm neonates by conventional feeding methods is still limited.

**Objective:**

This study aimed to systematically characterize gut microbiota variations across three feeding methods in neonates, with a specific focus on preterm neonates.

**Methods:**

This study included 265 neonates from the Hunan Children’s Hospital cohort, categorized by feeding methods into breast-fed (BF, *n* = 122), formula-fed (FF, *n* = 86), and mixed-fed (MF, *n* = 57) groups. Additionally, the analysis was further stratified into preterm and full-term neonates to investigate the relationship between feeding methods and gut microbiota. Gut microbiota composition was assessed using 16S rRNA gene sequencing of neonatal fecal samples. Microbial patterns associated with feeding methods were identified through MaAsLin2 analyses.

**Results:**

Unadjusted analyses showed that compared with the FF and MF groups, the BF group exhibited significantly higher relative abundances of *Bifidobacterium* (*p* = 0.018) and *Staphylococcus* (*p* = 0.039). In addition, the relative abundance of *Akkermansia* (*p* = 0.046) in the FF group was higher than that in the other two groups. MaAsLin2 analyses revealed the FF group had higher relative abundances of *Verrucomicrobiota* (Coef = 1.46, *p* = 0.001, FDR *q*-value = 0.055) and *Akkermansia* (Coef = 1.53, *p* = 0.001, FDR *q*-value = 0.040) than those in the BF group among all neonates, particularly preterm neonates. The BF neonates displayed enriched *Staphylococcus* (Coef = −1.47, *p* = 0.009, FDR *q*-value = 0.154) and depleted *Rothia* (Coef = 1.45*, p =* 0.004, FDR *q*-value = 0.121). The MF group showed higher *Streptococcus* abundance relative to the BF group (Coef = 0.95, *p* = 0.019, FDR *q*-value = 0.204). Functional prediction indicated potential group differences in propionate metabolism and degradation of valine, leucine, and isoleucine pathways, particularly in preterm neonates.

**Conclusion:**

Our study demonstrates an association between feeding methods and gut microbiota composition in neonates. The BF neonates exhibited increased relative abundance of *Staphylococcus* and decreased relative abundances of *Akkermansia* and *Rothia* compared with FF neonates. The MF group had higher relative abundance of *Streptococcus* than BF group. Furthermore, among preterm neonates, FF group was associated with a higher abundance of *Akkermansia* compared with BF group.

## 1 Introduction

Breast milk is considered the optimal source of neonatal nutrition (Meek and Noble, 2022). Exclusive breastfeeding is advocated by the World Health Organization for the initial 6 months of life. Breast milk provides essential nutrients to promote neonatal growth and development, alongside bioactive components that reduce the risk of illness (Ames et al., 2023; Gregory et al., 2016). However, feeding modalities differ widely among neonates, including breast-fed (BF), formula-fed (FF), and mixed-fed (MF), particularly for preterm neonates. On the one hand, the neonate’s physiological condition may render direct breastfeeding unsuitable; on the other hand, maternal capacity to breastfeed is influenced by numerous factors.

The establishment and maturation of the gut microbiota in early life are important to human health across the lifespan, and its community structure is closely linked to early nutritional exposure (Xiao and Zhao, 2023). Several factors, including feeding methods, mode of delivery, antibiotic exposure, gestational age, and birth weight, influence neonatal gut colonization. Neonatal feeding methods appear to be particularly crucial for shaping gut microbiota assembly (Li et al., 2020). The neonatal gut represents a newly established microbial ecosystem, where breast milk provides the nutritional foundation driving the colonization and succession of the early-life microbiota (Kiu et al., 2025). Studies from different cohorts have demonstrated that the diversity, taxonomic composition, and relative abundance of gut microbiota are impacted by feeding methods (Cai et al., 2019). Compared with non-breastfed neonates, BF neonates have higher abundance of *Bifidobacterium* in the gut (Davis et al., 2017).

Recent studies mostly focused on comparing the gut microbiota between BF and FF groups in full-term neonates, with limited attention to MF and preterm neonates. In addition, few systematic investigations have evaluated associations between three conventional feeding methods and neonatal gut microbiota, particularly in preterm neonates. Therefore, this study enrolled 265 neonates to characterize differences in gut microbiota composition across these three feeding groups, with a specific emphasis on preterm neonates. This study provides a preliminary foundation for future research by linking feeding methods with neonatal gut microbiota composition.

## 2 Materials and methods

### 2.1 Study subjects

A total of 481 neonates were recruited from August 1, 2018 to October 31, 2019 at Hunan Children’s Hospital in China. Neonates with a postnatal age >60 days at sampling, or those lacking complete gut microbiota or feeding data, were excluded. Ultimately, 265 neonates from the neonatal intensive care unit were included in the present study (Supplementary Figure S1). Participants were divided into BF, FF, and MF according to the definition of feeding methods. Additionally, based on a gestational age of 37 weeks, neonates were divided into preterm and full-term subgroups for analysis. This study was approved by the Medical Ethics Committee of Hunan Children’s Hospital (HCHL-2018-June 4), and written informed consent was obtained from all parental guardians.

### 2.2 Definition of feeding methods

**Figure 1:**
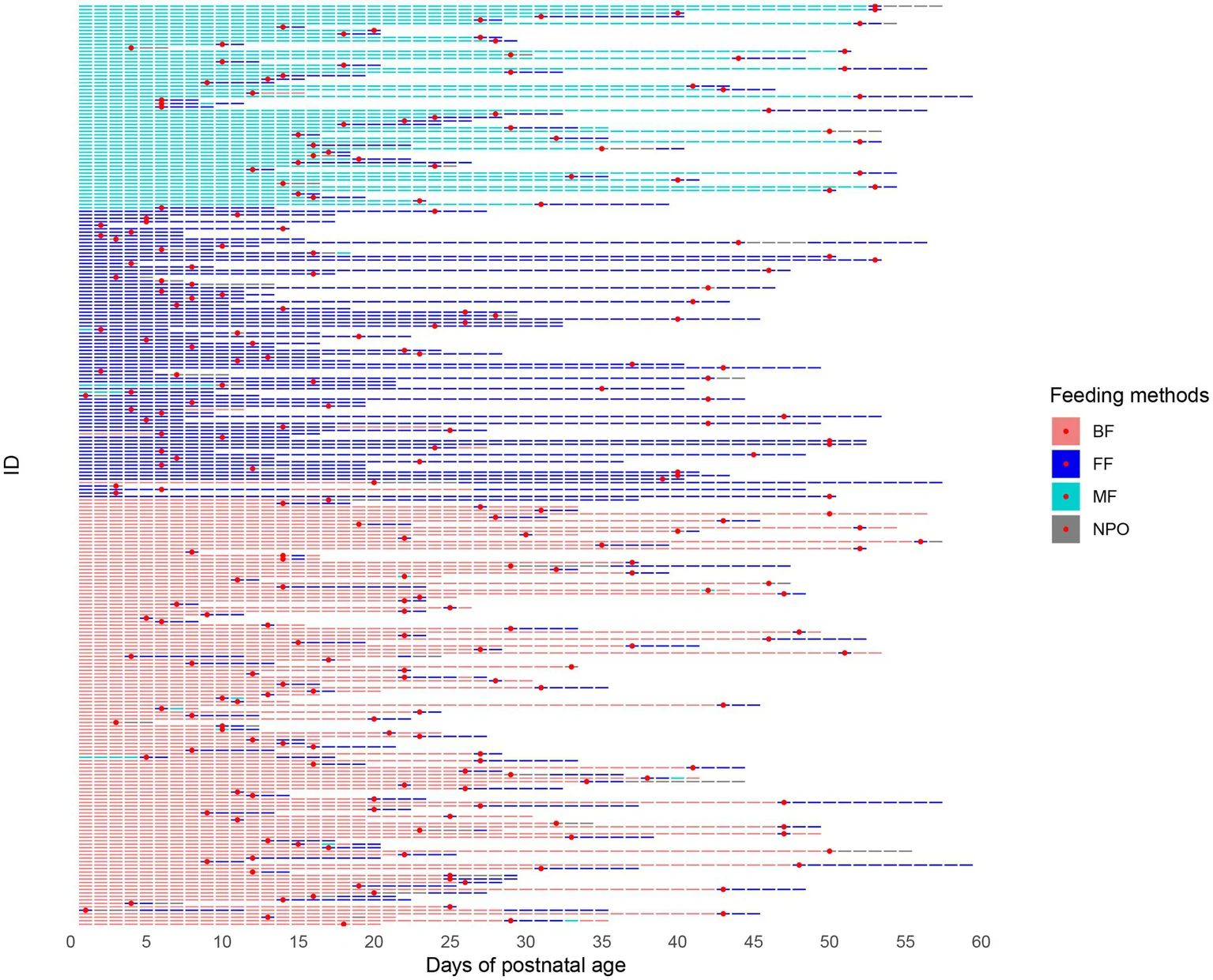
Feeding pattern distribution among 265 neonates before stool collection. The colors in the legend represent different feeding methods, and the red dots represent the admission time of the neonates. BF, breast-fed group; FF, formula-fed group; MF, mixed-fed group. NPO, no enteral feeding (parenteral nutrition).

Neonates were categorized into one of three feeding groups according to whether they were BF, FF, or MF. MF was defined as the receipt of both breast milk and formula on the same day. Feeding group was determined by the predominant feeding methods that accounted for >50% of total feeding days before sampling (Yang R. et al., 2019). No enteral feeding (NPO) was not regarded as a separate feeding-group category, but this period was retained in the feeding trajectory plot to represent the clinical history (Figure 1). Additionally, NPO days were included in the denominator when calculating the proportion of each feeding methods. The inclusion criteria are as follows: Complete and continuous feeding records were available to determine the proportion, duration, and sequence of breast milk and formula administration. The exclusion criteria are as follows: Neonates for whom the predominant feeding methods could not be determined according to the feeding group definition—that is, those who could not be assigned to any of the three groups (BF, FF, or MF) because none of the three feeding categories accounted for >50% of the total feeding days before stool collection—were excluded. In addition, those who received no enteral feeding at all or relied solely on parenteral nutrition prior to sampling were also excluded.

### 2.3 16S rRNA gene amplicon sequencing and processing

Fresh fecal samples were immediately aliquoted into sterile cryovials and transported to the laboratory under refrigeration within 2 h, followed by storage at −80 °C. Microbial profiling was conducted using 16S rRNA gene amplicon sequencing (Genesky Biotechnologies, Shanghai). Genomic DNA was extracted from stool samples using the QIAamp DNA Stool Mini Kit (Qiagen) per the manufacturer’s protocol. The concentration and purity of genomic DNA were detected by the Nanodrop 2000 and Qubit 3.0 Spectrophotometer, and the integrity was verified using agarose gel electrophoresis. The V4-V5 hypervariable region was amplified using the primers 515F (5′-GTG CCAGCMGCCGCGG-3′) and 907R (5′-CCGTCAATTC MTTT RAGTTT-3′). The sequencing of 16S rRNA was carried out on an Illumina NovaSeq 6,000 platform for generating 2 × 250 bp paired-end reads. QIIME2 was used to process the raw read sequences, with the cutadapt plugin applied to trim adapter and primer sequences. Quality filtering, denoising, merging, and chimera removal were performed using the DADA2 plugin with parameters maxEE = 2 and truncQ = 2, generating amplicon sequence variants (ASVs). ASVs matching the human reference genome (GRCh38) were identified by BLASTN alignment and removed (Walker et al., 2020). To mitigate background contamination, negative control samples were included throughout the experimental workflow, and contaminant ASVs were identified and removed using the Decontam R package based on a statistical classification framework. No additional filtering of low-abundance or low-prevalence ASVs was applied to the feature table prior to all gut microbiota analyses (Caruso et al., 2019). Taxonomic assignment of ASV representative sequences was performed using the SILVA 138.1 reference database with a pre-trained Naive Bayes classifier with a confidence threshold of 0.8 (Xiang et al., 2025).

### 2.4 Covariate assessment

Clinical information of neonates was retrieved from the electronic medical record system and via follow-up questionnaires. Seven covariates associated with the gut microbiota were included as potential confounders: sex (male/female), postnatal age (days), birth weight, gestational age, antibiotic exposure (yes/no), delivery mode (vaginal/cesarean) and pediatric diseases (respiratory/circulatory/ immune/others). Maternal disease was defined as any disease present during pregnancy.

### 2.5 Statistical analysis

Clinical characteristics were analyzed using descriptive statistics (SPSS 27.0). Normally distributed continuous variables were expressed as the mean ± standard deviation (X ± SD), while skewed continuous variables were reported as the median and interquartile range [M (P25–P75)]. Categorical variables were summarized as the absolute number and percentage [*n* (%)]. Differences among three groups were evaluated by analysis of variance (ANOVA), Kruskal-Wallis *H* test or Chi-square test/Fisher’s exact test, as appropriate.

All gut microbiota statistical analyses were performed using R (Version 4.5.0). Rarefaction was implemented with the R package *phyloseq*, with all samples randomly subsampled to the minimum sequencing depth observed across the dataset to ensure comparability of alpha and beta diversity metrics (Leontidou et al., 2023; Schloss, 2024). For α-diversity, community richness was measured using Chao1 and ACE indices, while diversity using Shannon and Simpson indices. For *β*-diversity, principal coordinate analysis (PCoA) was applied to visualize compositional differences in gut microbiota across groups, and PERMANOVA test was conducted to determine its significance. Redundancy analysis (RDA) was employed to explore the relationship between feeding methods and genus-level gut microbiota composition.

Microbiome Multivariate Associations with Linear Models (MaAsLin2) was used to identify associations between feeding methods and gut microbial taxa at the phylum and genus levels, with adjustment for clinical covariates. Taxa with a prevalence <10% were excluded. The taxa data were normalized to relative abundance by total-sum scaling, and pseudo-counts (half of the minimum relative abundance for each taxon) were added to zero counts prior to log transformation. Multiple testing was controlled using the Benjamini-Hochberg procedure, and associations with an FDR *q*-value < 0.25 were considered statistically significant (Vatanen et al., 2022; Ofordile et al., 2025). Based on the 16S rRNA gene sequence, the genetic function of gut microbiota in Kyoto Encyclopedia of Genes and Genomes (KEGG) was predicted by using Phylogenetic Investigation of Communities by Reconstruction of Unobserved States 2 (PICRUSt2). A sensitivity analysis was conducted by excluding samples collected within the first week of life, and the primary MaAsLin2 analyses were repeated accordingly. A *p*-value of less than 0.05 (two-tailed) was considered statistically significant.

## 3 Results

### 3.1 General characteristics

A total of 265 neonates (median postnatal age, 24 days) were included finally in the study. Among these neonates, 122 were BF, 86 were FF, and 57 were MF. The participants were 61.5% male, with a median birth weight of 2,450 g and a median gestational age of 36 weeks. Regarding sex, gestational age, antibiotic exposure, delivery mode, pediatric diseases or maternal health status, there were no discernible differences among the BF, FF, and MF groups (Table 1, *p* > 0.05). However, postnatal age, birth weight and maternal age differed significantly among the feeding groups (Table 1, *p* < 0.05). Neonates in the ≤7 days group had significantly shorter postnatal age relative to those in the >7 days group (Supplementary Table S3, *p* = 0.001). Additionally, feeding methods distribution differed significantly between the two groups (Supplementary Table S3, *p* = 0.001).

**Table 1:** Clinical characteristics of 265 neonates.

Characteristics	BF (*n* = 122)	FF (*n* = 86)	MF (*n* = 57)	H/χ^2^	*p*
*Neonatal characteristics*					
Sex, *n* (%)				3.824	0.148
Male	69 (56.60%)	60 (69.80%)	34 (59.60%)		
Female	53 (43.40%)	26 (30.20%)	23 (40.40%)		
Postnatal age(days), median (P25, P75)	25.0 (18.0, 37.0)	18.0 (10.8, 40.0)	28.0 (18.0, 44.5)	10.499	0.005
Birth weight(g), median (P25, P75)	2650.0 (1987.5, 3207.5)	2290.0 (1795.0, 2882.5)	2500.0 (2145.0, 3175.0)	7.307	0.026
Gestational age(weeks), median (P25, P75)	36.0 (33.8, 39.0)	35.0 (33.0, 36.0)	35.0 (34.0, 37.0)	5.583	0.061
Antibiotic exposure, *n* (%)				5.160	0.076
Yes	95 (77.9%)	74 (86.0%)	48 (84.2%)		
No	27 (22.1%)	12 (14.0%)	9 (15.8%)		
Pediatric disease				10.243	0.115
Respiratory system	84 (68.9%)	59 (68.6%)	38 (66.7%)		
Circulatory system	20 (16.4%)	4 (4.7%)	8 (14.0%)		
Immune system	7 (5.7%)	9 (10.5%)	3 (5.3%)		
Other systems	11 (9.0%)	14 (16.3%)	8 (14.0%)		
*Maternal characteristics*					
Maternal age (years), median (P25, P75)	29.0 (27.0, 31.0)	29.0 (27.0, 32.3)	32.0 (28.0, 35.5)	6.104	0.047
Delivery mode, *n* (%)				3.396	0.183
Vaginal	67 (54.9%)	45 (52.3%)	23 (40.4%)		
Cesarean	55 (45.1%)	41 (47.7%)	34 (59.6%)		
Maternal health status, *n* (%)				0.248	0.903
Yes	96 (78.7%)	70 (81.4%)	45 (78.9%)		
No	26 (21.3%)	16 (18.6%)	12 (21.1%)		

BF, breast-fed; FF, formula-fed; MF, mixed-fed. H, Kruskal-Wallis H statistic; χ^2^, Chi-square statistic. Maternal disease: any disease present during pregnancy.

### 3.2 The association of various feeding methods with gut microbiota

#### 3.2.1 Gut microbiota diversity, composition and differential taxa

**Figure 2:**
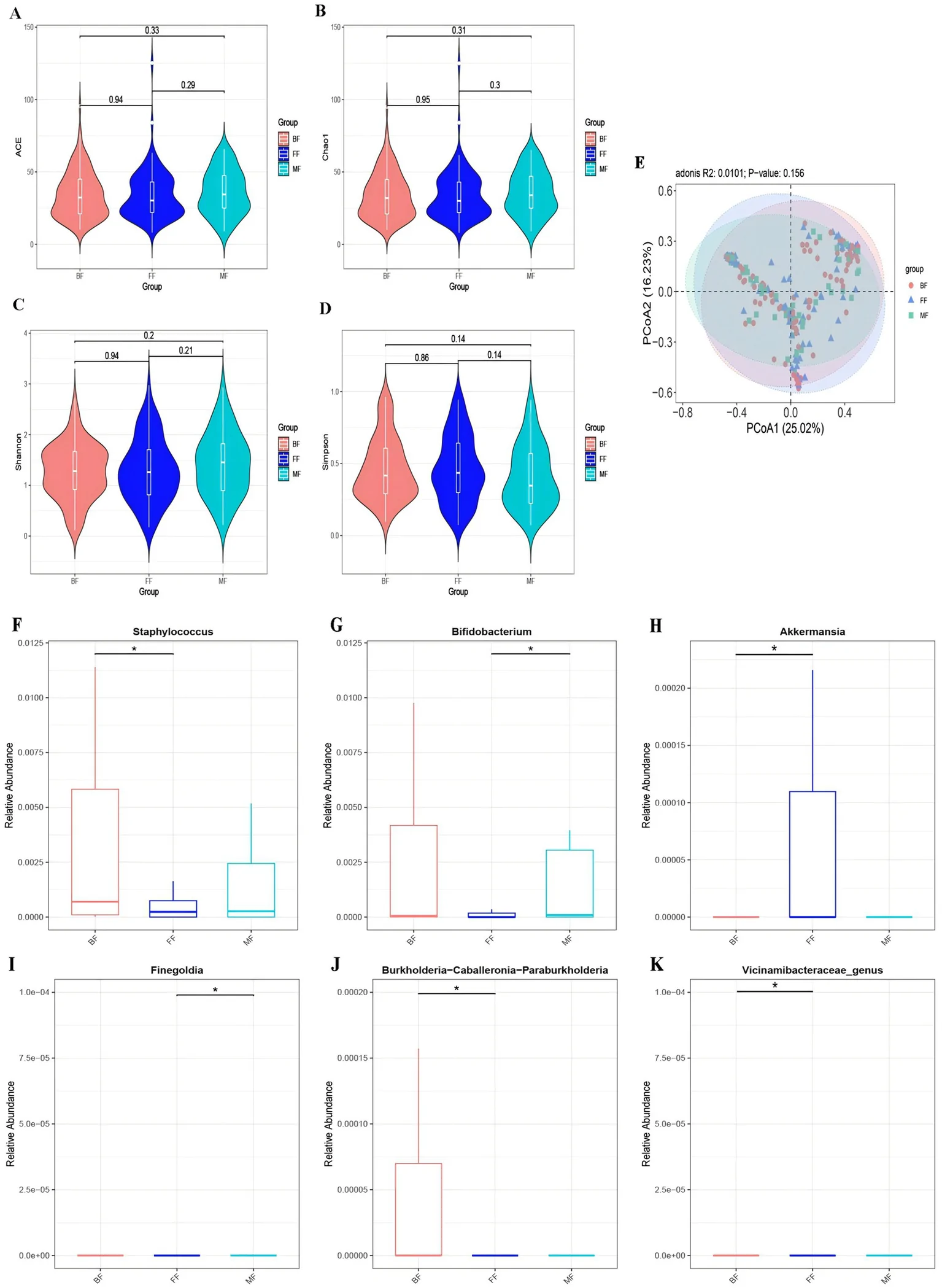
Gut microbiota diversity and relative abundance in the BF, FF, and MF groups in 265 neonates. **(A–D)** Comparison of the *α*-diversity indices among three groups. **(E)** PCoA among the three groups. **(F–K)** Differences in relative abundance at the genus level. *p < 0.05, **p < 0.01, ***p < 0.001, ****p < 0.0001. BF, breast-fed group; FF, formula-fed group; MF, mixed-fed group.

We obtained 17.66 million non-chimeric reads from the 265 samples via 16S rRNA sequencing, with a per-sample median read depth of 67,333 (Interquartile range: 65,759–68,798). The sufficiency and rationality of sampling were assessed using rarefaction curves and species accumulation curves for each sample in this study (Supplementary Figure S2). No significant differences in α-diversity indices (Figures 2A–D, *p* > 0.05) or *β*-diversity (Figure 2E, *p* > 0.05) were observed among the BF, FF, and MF groups.

According to the analysis of microbiota composition (Supplementary Figure S3), the predominant gut microbiota phyla in the three groups were *Firmicutes* (51.87, 50.85, 60.70%), *Proteobacteria* (42.54, 42.76, 31.25%), *Bacteroidota* (2.38, 3.21, 4.42%), and *Actinobacteriota* (2.74, 2.07, 3.28%). At the genus level, the predominant taxa were *Enterococcus* (28.03, 27.14, 33.19%), *Escherichia-Shigella* (19.83, 19.86, 17.59%), and *Streptococcus* (10.31, 11.90, 14.50%).

At the order level (Supplementary Figure S4A), there was an increasing trend in the relative abundance of *Bacteroidales* (0.023 vs. 0.035 vs. 0.043, *p* = 0.032) in BF, FF, and MF groups, while there was a decreasing trend in the relative abundance *Staphylococcales* (0.034 vs. 0.027 vs. 0.018, *p* = 0.047). The relative abundance of *Oscillospirales* and *Verrucomicrobiales* was higher in the FF group, but the lower abundance of *Bifidobacteriales* compared with the other groups (*p* < 0.05).

At the family level (Supplementary Figure S4B), *Bifidobacteriaceae* (0.019 vs. 0.003 vs. 0.015, *p* = 0.017), *Staphylococcaceae* (0.034 vs. 0.028 vs. 0.018, *p* = 0.015), *Burkholderiaceae* (0.0003 vs. 0.0001 vs. 0.0001, *p* = 0.008), *Oxalobacteraceae* (2.38E-06 vs. 5.36E-06 vs. 1.43E-06, *p* = 0.045), *Akkermansiaceae* (0.004 vs. 0.010 vs. 0.002, *p* = 0.046), *Listeriaceae* (0 vs. 1.75E-05 vs. 0, *p* = 0.015), and *UCG-010* (9.49E-06 vs. 6.99E-06 vs. 0, *p* = 0.046) were significantly different among the BF, FF, and MF groups. Moreover, the BF group harbored the highest relative abundances of *Bifidobacterium*, *Staphylococcaceae*, and *Burkholderiaceae*.

At the genus level (Figures 2F–K), significant abundance differences were detected for *Staphylococcus*, *Bifidobacterium*, *Akkermansia*, *Burkholderia-Caballeronia-Paraburkholderia*, *Finegoldia*, and *Vicinamibacteraceae*. Notably, the relative abundance trends of *Staphylococcus* (0.034 vs. 0.027 vs. 0.018, *p* = 0.039), *Bifidobacterium* (0.019 vs. 0.003 vs. 0.015, *p* = 0.015), and *Akkermansia* (0.004 vs. 0.010 vs. 0.002, *p* = 0.046) were consistent with the corresponding family-level patterns. In addition, the relative abundance of *Burkholderia-Caballeronia-Paraburkholderia* (2.78E-04 vs. 5.32E-05 vs. 5.66 E-05, *p* = 0.007) was higher in the BF group.

#### 3.2.2 Association between feeding methods and gut microbiota through RDA analysis and MaAsLin2

RDA revealed the relationship between microbiome composition and clinical characteristics. A significant association was observed between gut microbiota and feeding methods at the genus level (Supplementary Figure S5A). The MaAsLin2 analysis identified one phylum and five genera significantly associated with feeding methods (*p* < 0.05, FDR *q*-value< 0.25, Table 2). Compared with BF group, the FF group had higher relative abundance of *Verrucomicrobiota* (Coef = 1.46, *p* = 0.001, FDR *q*-value = 0.055), *Rothia* (Coef = 1.45, *p* = 0.004, FDR *q*-value = 0.121), and *Akkermansia* (Coef = 1.53, *p* = 0.001, FDR *q*-value = 0.040), along with reduced abundance of *Staphylococcus* (Coef = −1.47, *p* = 0.009, FDR *q*-value = 0.154) and *Burkholderia-Caballeronia-Paraburkholderia* (Coef = −0.90, *p* = 0.001, FDR *q*-value = 0.057). In addition, the MF group had lower relative abundance of *Burkholderia-Caballeronia-Paraburkholderia* (Coef = −0.76, *p* = 0.012, FDR *q*-value = 0.183) compared to the BF group, and higher relative abundance of *Streptococcus* (Coef = 0.95, *p* = 0.019, FDR *q*-value = 0.204). Furthermore, sensitivity analysis was performed by excluding samples from the first week of life, and the main associations between feeding methods and gut microbiota remained robust (Supplementary Table S4). In addition to feeding-related associations, the MaAsLin2 identified significant associations between several covariates and specific taxa (Supplementary Figure S5B). Compared with neonates without antibiotic exposure, those exposed to antibiotics had higher relative abundance of *Enterococcus* (Coef = 1.803, *p* < 0.001, FDR *q*-value = 0.051). The relative abundances of *Bacteroides* (Coef = −1.245, *p* = 0.016, FDR *q*-value = 0.204) and *Parabacteroides* (Coef = −0.118, *p* = 0.008, FDR *q*-value = 0.148) were lower in neonates delivered by cesarean section than in those delivered vaginally.

**Table 2:** Relationship between gut microbiota (phylum and genus) and different feeding methods of 265 neonates.

Phylum	Genus	Value	Coef	SE	*p*	FDR *q*-value
*Verrucomicrobiota*	*—*	2	1.46	0.434	0.001	0.055
*Verrucomicrobiota*	*Akkermansia*	2	1.53	0.434	0.001	0.040
*Proteobacteria*	*Burkholderia. Caballeronia. Paraburkholderia*	2	−0.90	0.278	0.001	0.057
*Actinobacteriota*	*Rothia*	2	1.45	0.507	0.004	0.121
*Firmicutes*	*Staphylococcus*	2	−1.47	0.560	0.009	0.154
*Firmicutes*	*Streptococcus*	3	0.95	0.400	0.019	0.204
*Proteobacteria*	*Burkholderia. Caballeronia. Paraburkholderia*	3	−0.76	0.303	0.012	0.183

Associations were analyzed using MaAsLin2. Neonates feeding methods covariate set: sex (male/female), postnatal age (days), birth weight, gestational age, antibiotic exposure (yes/no), delivery mode (vaginal/cesarean) and pediatric disease (respiratory/circulatory/ immune/others). —: data unavailable, Value: 1 = BF group (reference group); 2 = FF group; 3 = MF. group, it reports the correlation of classification features. Coef: coefficient, Coef indicates the fold change in relative abundance (2^Coef) from the reference group to the comparison group specified in the “value” column. SE: standard error, MaAsLin2: microbiome multivariate associations with linear models.

#### 3.2.3 Analysis of KEGG’S metabolic pathway

**Figure 3:**
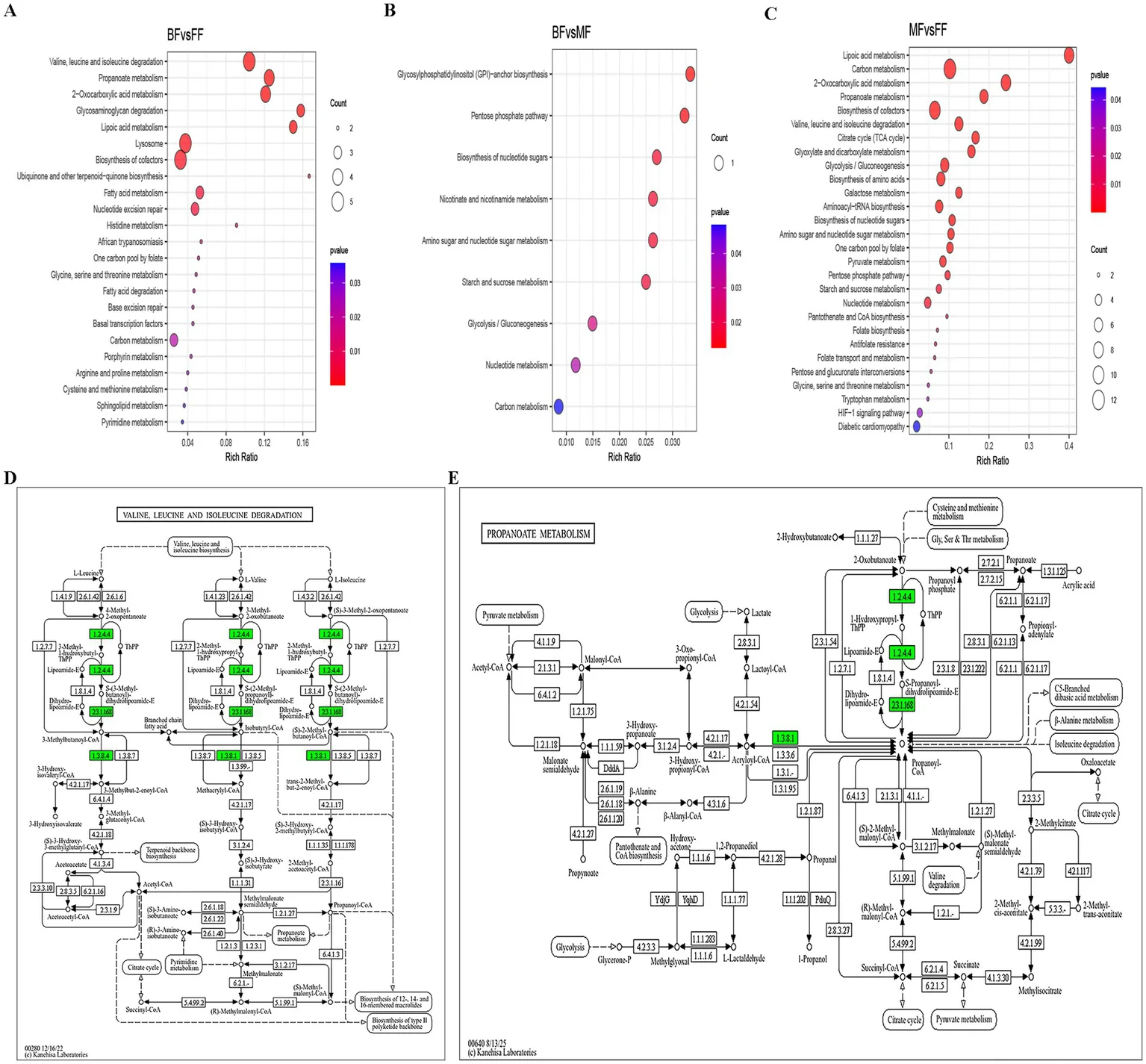
Difference analysis of metabolic pathways in 265 neonates. **(A–C)** Metabolic pathway analysis based on pairwise comparisons among the three groups. The color represents the significance level, while the point size reflects the number of genes. **(D)** Differences in valine, leucine and isoleucine degradation between the BF and FF groups. **(E)** Differences in propanoate metabolism between the BF and FF groups. Red: enriched in FF group; Green: nriched in BF group.

Pairwise comparative analysis was performed to screen differentially enriched KEGG pathways among the BF, FF, and MF groups based on PICRUSt2-predicted functional profiles. Specifically, 23 pathways differed significantly between the BF and FF groups, 9 between the BF and MF groups, and 28 between the FF and MF groups (Figures 3A–C). Degradation of valine, leucine, and isoleucine and propanoate metabolism were identified as the main contributors to the intergroup differences (Figures 3D–E).

### 3.3 The association of various feeding methods with gut microbiota in preterm and full-term neonates

#### 3.3.1 General characteristics

The three groups of preterm neonates differed significantly in postnatal age, whereas no other variables exhibited statistically significant differences across the three feeding groups in either preterm or full-term neonates (Supplementary Tables S1 and S2).

#### 3.3.2 Gut microbiota diversity, composition and differential taxa

**Figure 4:**
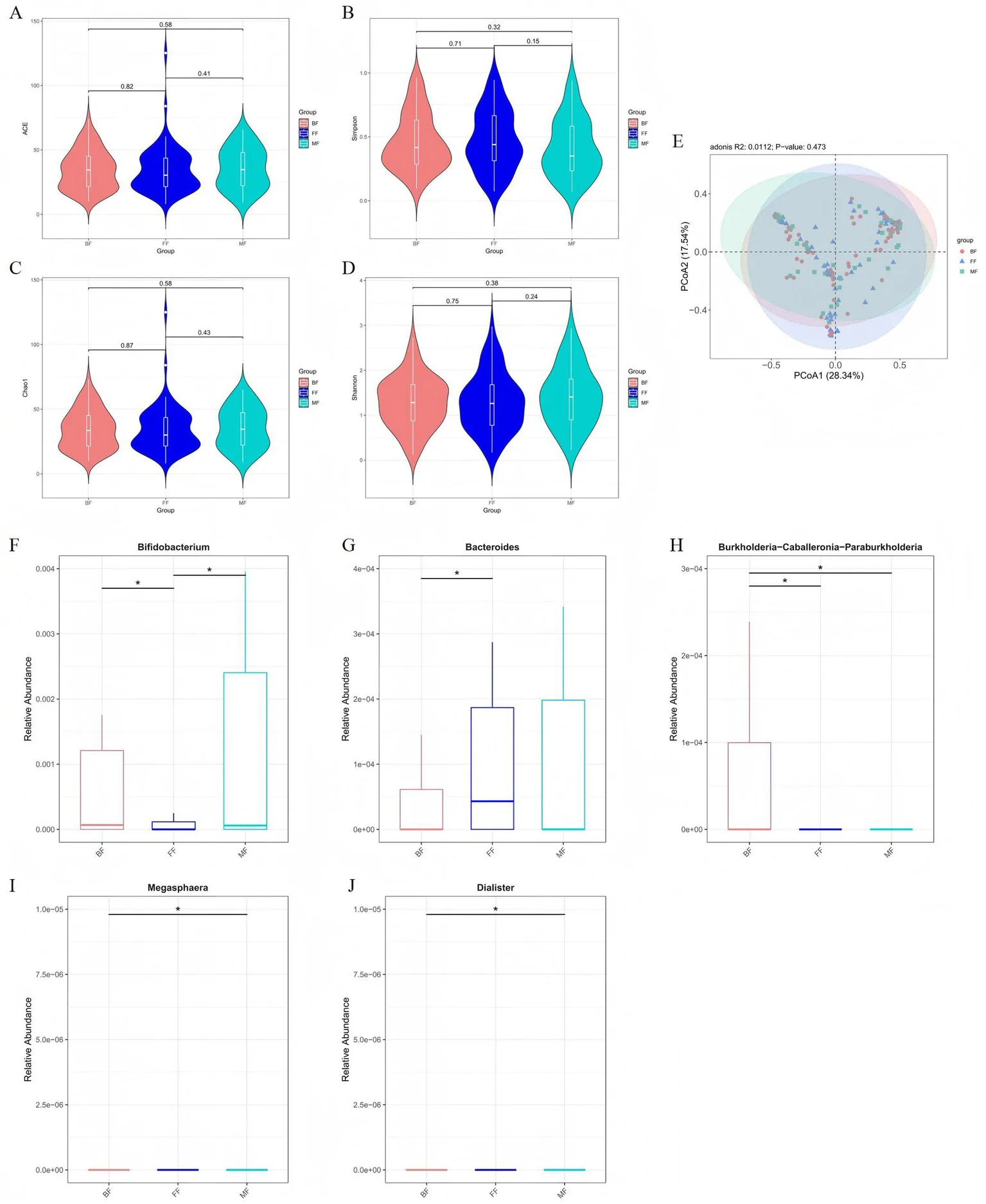
Gut microbiota diversity and relative abundance in the BF, FF, and MF groups in 177 preterm neonates. **(A–D)** Comparison of the α-diversity indices among three groups. **(E)** PCoA among the three groups. **(F–J)** Differences in relative abundance at the genus level. *p < 0.05, **p < 0.01, ***p < 0.001, ****p < 0.0001. BF, breast-fed group; FF, formula-fed group; MF, mixed-fed group.

There were no significant differences in *α* and *β* diversity of the gut microbiota across the three feeding methods in either preterm or full-term neonates (Figures 4A–E; Supplementary Figures S8A–E).

In both preterm and full-term neonates, the core compositional structure of the gut microbiota remained stable across feeding groups (Supplementary Figure S6). At the phylum level, the predominant taxa were *Firmicutes* (preterm: 49.59, 45.96, 54.68%; full-term: 65.18, 55.41, 77.5%), *Proteobacteria* (preterm: 46.84, 47.37, 37.79%; full-term: 25.62, 37.81, 12.92%), *Bacteroidota* (preterm: 0.77, 3.17, 4.47%; full-term: 7.14, 2.92, 4.29%), and *Actinobacteriota* (preterm: 2.25, 2.06, 2.59%; full-term: 1.97, 3.41, 5.20%). At the genus level, the dominant taxa were *Enterococcus* (preterm: 27.81, 23.92, 33.02%; full-term: 37.11, 28.53, 33.64%), *Escherichia-Shigella* (preterm: 25.03, 22.90, 22.05%; full-term: 9.00, 13.62, 5.13%), and *Streptococcus* (preterm: 6.39, 9.94, 8.60%; full-term: 17.77, 15.43, 31.01%).

In preterm neonates, significant differences were found across the feeding groups at the family level for *Bifidobacteriaceae*, *Bacteroidaceae*, *Burkholderiaceae* (*p* < 0.05; Supplementary Figure S7), and at the genus level for *Bifidobacterium*, *Bacteroides*, *Burkholderia-Caballeronia-Paraburkholderia* (*p* < 0.05, Figures 4F–J). Pairwise analysis revealed that the relative abundances of *Bifidobacterium* and *Burkholderia-Caballeronia-Paraburkholderia* were higher in the BF group compared to the FF group. Conversely, the relative abundance of *Bacteroides* was lower in the BF group.

In full-term neonates, significant intergroup differences were observed for *Proteobacteria*, *Enterobacterales*, *Family_XI*, *[Clostridium] _ Innocumum _ Group*, *Microlunatus* and *Paenibacillus* (*p* < 0.05, Supplementary Figures S8F–K). The MF group had higher levels of *Microlunatus* and *Paenibacillus*, whereas the FF group had higher levels of *[Clostridium] innocuum Group*.

#### 3.3.3 Association between feeding methods and gut microbiota through MaAslin2

After covariate adjustment via MaAsLin2, significant associations between feeding methods and gut microbiota were identified in preterm neonates (Table 3). However, no significant associations were identified in full-term neonates. Specifically, compared with the BF group, the FF group exhibited higher relative abundance of *Verrucomicrobiota* (Coef = 1.70, *p* = 0.003, FDR *q*-value = 0.109), *Akkermansia* (Coef = 1.74, *p* = 0.002, FDR *q*-value = 0.104), and *Bacteroides* (Coef = 1.77, *p* = 0.009, FDR *q*-value = 0.229), accompanied by lower abundance of *Burkholderia-Caballeronia-Paraburkholderia* (Coef = −0.95, *p* = 0.004, FDR *q*-value = 0.172) in preterm neonates. Among preterm neonates, the MF group had lower abundance of *Burkholderia-Caballeronia-Paraburkholderia* (Coef = −0.96, *p* = 0.010, FDR *q*-value = 0.229) relative to the BF group. The key correlations between feeding methods and gut microbiota in preterm neonates persisted after a sensitivity analysis excluding samples from the first week of life (Supplementary Table S4).

**Table 3:** Relationship between gut microbiota (phylum and genus) and different feeding methods of preterm neonates.

Phylum	Genus	Value	Coef	SE	*p*	FDR *q*-value
*Verrucomicrobiota*	—	2	1.70	0.563	0.003	0.109
*Verrucomicrobiota*	*Akkermansia*	2	1.74	0.564	0.002	0.104
*Proteobacteria*	*Burkholderia. Caballeronia. Paraburkholderia*	2	−0.95	0.327	0.004	0.172
*Bacteroidota*	*Bacteroides*	2	1.77	0.673	0.009	0.229
*Proteobacteria*	*Burkholderia. Caballeronia. Paraburkholderia*	3	−0.96	0.364	0.010	0.229

Associations were analyzed using MaAsLin2. Neonates feeding methods covariate set: sex (male/female), postnatal age (days), birth weight, gestational age, antibiotic exposure (yes/no), delivery mode (vaginal/cesarean) and pediatric disease (respiratory/circulatory/ immune/others). —: data unavailable, Value: 1 = BF group (reference group); 2 = FF group; 3 = MF. group, it reports the correlation of classification features. Coef: coefficient, Coef indicates the fold change in relative abundance (2^Coef^) from the reference group to the comparison group specified in the “value” column. SE: standard error, MaAsLin2: microbiome multivariate associations with linear models.

#### 3.3.4 Analysis of KEGG’S metabolic pathway

**Figure 5:**
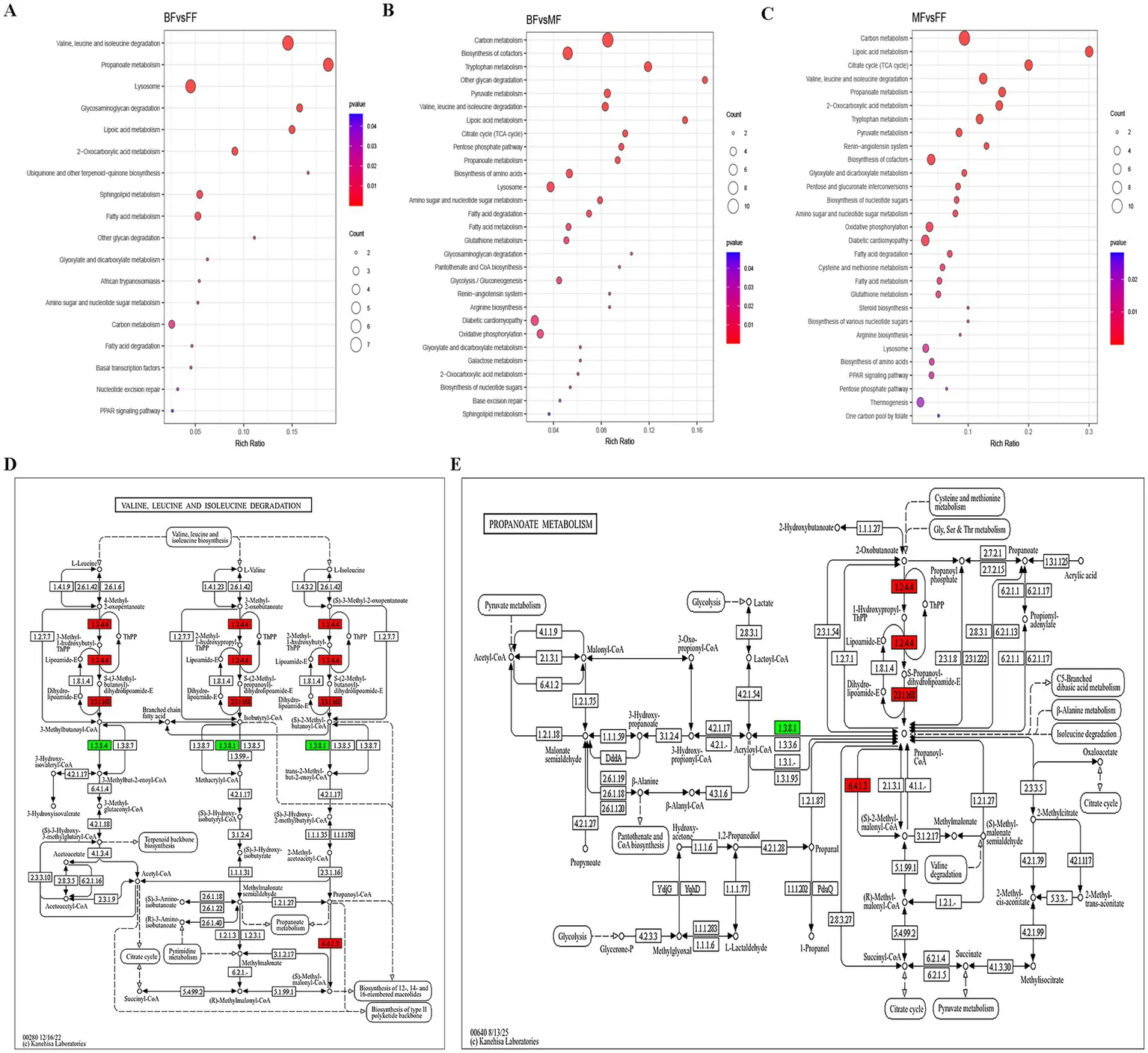
Difference analysis of metabolic pathways in 177 preterm neonates. **(A–C)** Metabolic pathway analysis based on pairwise comparisons among the three groups. The color represents the significance level, while the point size reflects the number of genes. **(D)** Differences in valine, leucine and isoleucine degradation between the BF and FF groups. **(E)** Differences in propanoate metabolism between the BF and FF groups. Red: Enriched in FF group; Green: Enriched in BF group.

In preterm neonates, there were statistically discernible differences in 18 pathways between BF and FF groups, 29 pathways between BF and MF groups, and 29 pathways between FF and MF groups (Figures 5A–C). The propionate metabolism and the degradation of valine, leucine, and isoleucine constituted the main pathways driving functional differences across the three feeding groups (Figures 5D–E).

In full-term neonates, inter-group differences in 13 pathways between BF and FF, 23 between BF and MF, and 34 between FF and MF (Supplementary Figures S9A–C). The lipoic acid metabolism and citrate cycle were the primary contributors to intergroup functional discrepancies (Supplementary Figures S9D–E).

## 4 Discussion

Findings from the present study showed feeding methods were associated with the gut microbiota. Our results showed no discernible variations in gut microbial diversity among the feeding groups. A previous study has reported that BF neonates have lower microbial diversity than non-breastfed neonates, a difference that may be explained by the selective enrichment of particular taxa via human milk oligosaccharides (Chong et al., 2022). The inconsistent findings observed in the present study may be attributed to the confounding of heterogeneous feeding histories within each group, which diluted intergroup differences in the gut microbiota.

In the current study, *Firmicutes*, *Proteobacteria*, *Bacteroidota*, and *Actinobacteriota* were identified as the predominant phyla across all feeding groups in neonates, consistent with previous studies demonstrating that these four phyla constitute the core gut microbiota in early infancy (Yang B. et al., 2019; Li et al., 2022). Our findings further support that the colonization of these phyla is a characteristic of the neonatal gut, and feeding methods may be primarily associated with their relative contributions rather than their overall presence. MaAsLin2 analysis revealed FF neonates had higher relative abundance of *Verrucomicrobiota* than BF neonates. The increase of *Verrucomicrobiota* in the FF group appeared to be mainly driven by the enrichment of *Akkermansia* at the genus level.

At the genus level, *Bifidobacteria* was significantly higher in the MF group than in the FF group among all neonates. Within the preterm subgroup, the BF group exhibited a significantly higher abundance than the FF group. The elevated relative abundance of *Bifidobacterium* in the BF and MF groups may be attributed to the high abundance of oligosaccharides in breast milk, which promote the proliferation of the genus (Davis et al., 2022). Importantly, *Bifidobacterium* can produce aromatic lactic acid within the neonatal gut, and several studies have indicated that these microbial metabolites may modulate immune function in early life (Laursen et al., 2021). This immunomodulatory potential is supported by an animal study demonstrating that in rats pretreated with *Bifidobacterium animalis subsp. lactis*, the intervention promoted the balance of T lymphocyte subsets in the spleen and thymus, as well as neurological development and neuroimmune balance in the brain (Lin et al., 2024). However, the overall relative abundance of *Bifidobacterium* observed in our study was lower than that previously reported. This inconsistency may stem from several factors, including the high proportion of preterm neonates in the cohort and potential geographical or ethnic differences (Lu et al., 2023).

The current study found that the relative abundance of *Streptococcus* was enriched in the MF group compared with the BF group after adjustment for variables. A longitudinal study analyzed fecal microbiota from 39 neonates and reported that exclusive breastfeeding during the first month of life was closely associated with a significantly lower relative abundance of *Streptococcus* (Fehr et al., 2020). Previous studies have shown that the level of *Streptococcus* in the intestine of patients with type 1 diabetes is high (Levin et al., 2016). *Streptococcus* is reported to be associated with other adverse effects. However, the mechanistic role of *Streptococcus* in the neonatal gut remains poorly characterized. We observed that the FF group exhibited elevated relative abundance of *Rothia* compared with the BF group after adjustment for potential confounders. Few studies have investigated the association between feeding methods and *Rothia* colonization in the neonatal gut. An oral microbiome study of infants revealed that the proportion of *Rothia* in MF infants was higher than that in exclusively breastfeeding infants, suggesting that feeding methods may affect the colonization of *Rothia* through oral-intestinal route (Sánchez-Morán et al., 2026). Future studies are needed to employ metagenomic sequencing to clarify the species-level composition and function of *Rothia* enriched by different feeding methods, and to elucidate the ecological role of this genus.

In this study, the relative abundance of *Staphylococcus* was higher in the BF group, followed by the FF and MF groups. This distribution is biologically reasonable. In addition to microbial exposure via breast milk, breastfeeding facilitates more extensive mother-neonate skin-to-skin contact than formula feeding (Fehr et al., 2020). Thus, bacteria originating from maternal skin and mammary ducts, including *Staphylococcus*, are more likely to be transmitted to BF neonates. By comparison, such exposure is probably lower in FF neonates than BF neonates (Pannaraj et al., 2017). MaAsLin2 analysis revealed that, among all neonates (particularly preterm neonates), the relative abundance of *Akkermansia* was significantly higher in the FF group than in the BF group. This observation has rarely been documented in previous studies (Odiase et al., 2023). *Akkermansia muciniphila* has attracted considerable attention in recent research on host health, and growing studies have reported its beneficial effects (Panzetta and Valdivia, 2024; Hughes et al., 2025). However, prior studies have shown that this genus has a considerable species diversity, with at least 31 species that differ significantly in immunoregulatory and metabolic activities (Hughes et al., 2025; Molteni et al., 2025). The marked differences in components between formula and breast milk may provide distinct growth substrates and ecological niches for different *Akkermansia* species, potentially accounting for the relative enrichment of these taxa in the FF group. Metagenomic sequencing could provide deeper insights into the species-level composition and strain-specific functional potential of *Akkermansia*. However, given the low overall prevalence of *Akkermansia* (27.2%) and it’s zero median relative abundance in all groups, this finding should be interpreted cautiously, as the difference may be driven by a few positive samples.

To assess the robustness of our main findings, we repeated the MaAsLin2 analysis after excluding samples collected during the first postnatal week. The results showed that the core associations between feeding methods and gut microbiota remained unchanged, which confirmed that these findings were not driven by the first-week samples.

Significant variations in the predicted pathways associated with the metabolism of propanoate and the degradation of valine, leucine, and isoleucine were found in our study among the BF, FF, and MF groups in total and preterm neonates. Valine, leucine, and isoleucine are all branched-chain amino acids (BCAAs) and serve important physiological functions in the human body. Through specific signaling networks, BCAA function as signaling molecules that affect immune response, gastrointestinal health, lipid and protein synthesis, and glucose metabolism (Dimou et al., 2022; Nie et al., 2018). Notably, our PICRUSt2-based predictions showed an enrichment of BCAA degradation pathway in the BF group, suggesting a potential association between breastfeeding and BCAA-related functional capacity of the gut microbiota. Additionally, our results revealed differences in the predicted propanoate-metabolizing potential of the gut microbiota across feeding groups. Previous observational studies have reported that children with higher fecal short-chain fatty acid, especially butyrate and propionate, exhibit decreased susceptibility to food and airborne allergens and a lower risk of subsequent allergic diseases (Roduit et al., 2019). The prediction of 16S rRNA function only reflects the potential metabolic capabilities of the microbial community and is less reliable than the metabolic phenotype measured by metabolomics. Future studies integrating fecal metabolomics are needed to define gut metabolite signatures.

This study had the following limitations. Firstly, recruitment from a hospital primarily caring for preterm neonates introduced an inherent selection bias, leading to an overrepresentation of preterm neonates in the final sample, which may limit the generalizability of our findings to neonatal populations. Secondly, the study’s reliance on a single time point sampling limited our ability to assess temporal variations in neonatal gut microbiota associated with different feeding methods. Thirdly, potential misclassification in the categorization of feeding methods, as well as heterogeneity in the feeding trajectories among feeding groups cannot be ruled out. Such within-group heterogeneity across feeding categories may have attenuated true microbiota differences and may partly explain the lack of clearer separation among groups. Fourthly, data on antibiotic timing and duration, as well as other potential clinical variables associated with gut microbiota, were not available, which may limit the interpretation of our findings. Finally, the V4-V5 region used in this study may underestimate the relative abundance of some taxa, particularly *Bifidobacterium*. Future research should consider using shotgun metagenomic sequencing for fecal samples of preterm neonates.

## 5 Conclusion

The present study revealed an association between feeding methods and gut microbiota composition in neonates, particularly preterm neonates. BF neonates exhibited an increase in the relative abundance of *Staphylococcus* and a decrease in the relative abundance of *Akkermansia* and *Rothia* compared with FF neonates. The MF group had higher relative abundance of *Streptococcus* than the BF group. Among preterm neonates, the FF group was associated with higher abundance of *Akkermansia* compared with the BF group. Further validation of these findings requires large-scale, prospective population-based investigations as well as experimental studies.

## Data Availability

The raw sequencing data are available in the NCBI Sequence Read Archive (SRA) under BioProject PRJNA1290671. Other data will be made available on request.
