# Supplementary material for: Association of feeding methods in early life with gut microbiota in neonates
Source: Front Microbiol. 2026 Sep 11;17:1921787. doi: 10.3389/fmicb.2026.1921787 (PMC13575991; doi:10.3389/fmicb.2026.1921787)
Supplement: Supplementary file 1 [file Supplementary_file_1.DOCX]

**Supplementary appendix**

**Contents**

Table S1 Clinical characteristics of 177 preterm neonates.

Table S2 Clinical characteristics of 88 full-term neonates.

Table S3 Clinical characteristics of neonates within and after the first week of life.

Table S4 Relationship between gut microbiota (phylum and genus) and different feeding methods after excluding neonates with postnatal age ≤7 days.

Figure S1 Flow diagram of participants selection.

Figure S2 Rarefaction and Species accumulation curves.

Figure S3 Relative abundance of gut microbiota in BF, FF and MF groups of 265 neonates at the level of phylum and genus.

Figure S4 The relative abundance of gut microbiota in the BF, FF, and MF groups among 265 neonates.

Figure S5 Associations between feeding methods and gut microbiota were analyzed using RDA and MaAslin2 analysis in 265 neonates.

Figure S6 Relative abundance of gut microbiota in BF, FF and MF groups of premature and full-term neonates at the level of phylum and genus.

Figure S7 The relative abundance of gut microbiota in the BF, FF, and MF groups among 177 preterm neonates.

Figure S8 Gut microbiota diversity and relative abundance in the BF, FF, and MF groups in 88 full-term neonates.

Figure S9 Difference analysis of metabolic pathways in 88 full-term neonates.

**Table S1**

**Clinical characteristics of 177 preterm neonates.**

| **Characteristics** | **BF(n=68)** | **FF(n=67)** | **MF(n=42)** | **H/**χ² | ***P*** |
| --- | --- | --- | --- | --- | --- |
| **Neonatal Characteristics** |  |  |  |  |  |
| Sex, n (%) |  |  |  | 4.218 | 0.124 |
| Male | 35(51.50%) | 46(68.70%) | 26(61.90%) |  |  |
| Female | 33(48.50%) | 21(31.30%) | 16(38.10%) |  |  |
| Postnatal age(days), median (P25, P75) | 33.0(22.3,45.0) | 19.0(12.0,44.0) | 35.0(21.3,51.5) | 11.325 | 0.003 |
| Birth weight(g), median (P25, P75) | 2200.0(1657.5,2587.5) | 2050.0(1700.0,2500.0) | 2400.0(1987.5,2612.5) | 3.924 | 0.141 |
| Gestational age(weeks), median (P25, P75) | 34.0(32.0,35.8) | 35.0(32.0,35.0) | 34.0(33.0,36.0) | 1.712 | 0.425 |
| Antibiotic exposure, n (%) |  |  |  | 0.637 | 0.727 |
| Yes | 59(86.8%) | 61(91.0%) | 37(88.1%) |  |  |
| No | 9(13.2%) | 6(9.0%) | 5(11.9%) |  |  |
| Pediatric disease |  |  |  | 5.355 | 0.499 |
| Respiratory system | 48(70.6%) | 47(70.1%) | 29(69.0%) |  |  |
| Circulatory system | 7(10.3%) | 2(3.0%) | 5(11.9%) |  |  |
| Immune system | 6(8.8%) | 7(10.4%) | 2(4.8%) |  |  |
| Other systems | 7(10.3%) | 11(16.4%) | 6(14.3%) |  |  |
| **Maternal Characteristics** |  |  |  |  |  |
| Maternal age (years)，median (P25, P75) | 29.0(27.0,31.0) | 29.0(27.0,32.0) | 31.0(28.0,35.0) | 5.008 | 0.082 |
| Delivery mode, n (%) |  |  |  | 2.853 | 0.247 |
| Vaginal | 33(48.50%) | 32(47.80%) | 14(33.30%) |  |  |
| Cesarean | 35(51.50%) | 35(52.20%) | 28(66.70%) |  |  |
| Maternal health status, n (%) |  |  |  | 1.360 | 0.518 |
| Yes | 49(72.10%) | 54(80.60%) | 32(76.20%) |  |  |
| No | 19(27.90%) | 13(19.40%) | 10(23.80%) |  |  |

BF, breast-fed; FF, formula-fed; MF, the mixed-fed. H, Kruskal‑Wallis H statistic; χ², Chi‑square statistic. Maternal disease: any disease present during pregnancy.

**Table S2**

**Clinical characteristics of 88 full-term neonates.**

| **Characteristics** | **BF(n=54)** | **FF(n=19)** | **MF(n=15)** | **H/χ²** | ***P*** |
| --- | --- | --- | --- | --- | --- |
| **Neonatal Characteristics** |  |  |  |  |  |
| Sex, n (%) |  |  |  | 1.528 | 0.427 |
| Male | 34(63.00) | 14(73.70) | 8(53.30) |  |  |
| Female | 20(37.00) | 5(26.30) | 7(46.70) |  |  |
| Postnatal age(days), median (P25, P75) | 19.5(13.0,27.0) | 13.0(8.0,22.0) | 20.0(12.0,26.0) | 5.786 | 0.055 |
| Birth weight(g), median (P25, P75) | 3250.0(2900.0,3500.0) | 3300.0(2900.0,3590.0) | 3350.0(2900.0,3550.0) | 0.700 | 0.705 |
| Gestational age(weeks), median (P25, P75) | 39.0(38.0,39.0) | 39.0(38.0,40.0) | 39.0(38.0,40.0) | 0.486 | 0.784 |
| Antibiotic exposure, n (%) |  |  |  | 0.241 | 0.886 |
| Yes | 36(66.7%) | 13(68.4%) | 11(73.3%) |  |  |
| No | 18(33.3%) | 6(31.6%) | 4(26.7%) |  |  |
| Pediatric disease |  |  |  | 4.995 | 0.405 |
| Respiratory system | 36(66.7%) | 12(63.2%) | 9(60.0%) |  |  |
| Circulatory system | 13(24.1%) | 2(10.5%) | 3(20.0%) |  |  |
| Immune system | 1(1.9%) | 2(10.5%) | 1(6.7%) |  |  |
| Other systems | 4(7.4%) | 3(15.8%) | 2(13.3%) |  |  |
| **Maternal Characteristics** |  |  |  |  |  |
| Maternal age (years)，median (P25, P75) | 29.0(28.0,32.0) | 29.0(26.0,33.0) | 32.0(27.0,36.0) | 2.708 | 0.258 |
| Delivery mode, n (%) |  |  |  | 0.284 | 0.904 |
| Vaginal | 34(63.00) | 13(68.40) | 9(60.00) |  |  |
| Cesarean | 20(37.00) | 6(31.60) | 6(40.00) |  |  |
| Maternal health status, n (%) |  |  |  | 0.305 | 1.000 |
| Yes | 47(87.00) | 16(84.20) | 13(86.70) |  |  |
| No | 7(13.00) | 3(15.80) | 2(13.30) |  |  |

BF, breast-fed; FF, formula-fed; MF, the mixed-fed. H, Kruskal‑Wallis H statistic; χ², Chi‑square statistic. Maternal disease: any disease present during pregnancy.

**Table S3**

**Clinical characteristics of neonates within and after the first week of life**

| **Characters** | **≤7 days (n=16)** | **>7 days (n=249)** | **U/χ²** | ***P*** |
| --- | --- | --- | --- | --- |
| Sex, n (%) |  |  | 2.803 | 0.094 |
| Male | 13(81.25) | 150(60.24) |  |  |
| Female | 3(18.75) | 99(39.76) |  |  |
| Postnatal age(days), median (P25, P75) | 6.0(4.5,7) | 26.0(17.0,40) | 3984 | 0.001 |
| Birth weight(g), median (P25, P75) | 2650.0(2150.0,3287.5) | 2440.0(1900.0,3100.0) | 1766 | 0.447 |
| Sampling weight(g), (P25, P75) | 2515.0(2075.0,3242.5) | 2900.0(2245.0,3550.0) | 2490 | 0.094 |
| Gestational age(weeks), median (P25, P75) | 36.5(35.0,39.0) | 36.0(33.0,38.0) | 1528 | 0.117 |
| Antibiotic exposure, n (%) |  |  | 1.894 | 0.169 |
| Yes | 13(81.25) | 230(92.37) |  |  |
| No | 3(18.75) | 19(7.63) |  |  |
| Delivery mode, n (%) |  |  | 0.353 | 0.553 |
| Vaginal | 7(43.75) | 128(51.41) |  |  |
| Cesarean | 9(56.25) | 121(48.59) |  |  |
| Feeding method, n (%) |  |  | 14.096 | 0.001 |
| BF | 3(18.75) | 119(47.79) |  |  |
| FF | 12(75.00) | 74(29.72) |  |  |
| MF | 1(6.25) | 56(22.49) |  |  |
| Preterm birth, n (%) |  |  | 2.165 | 0.141 |
| Yes | 8(50.00) | 169(67.87) |  |  |
| No | 8(50.00) | 80(32.13) |  |  |

BF, breast-fed; FF, formula-fed; MF, the mixed-fed. U, Mann-Whitney U statistic; χ², Chi‑square statistic. Sampling weight: body weight at stool collection.

**Table S4**

**Relationship between gut microbiota (phylum and genus) and different feeding methods after excluding neonates with postnatal age ≤7 days.**

| **Neonate types** | **N** | **Phylum** | **Genus** | **Value** | **Coef** | **SE** | ***P*** | **FDR *q*-value** |
| --- | --- | --- | --- | --- | --- | --- | --- | --- |
| Total | 249 | *Verrucomicrobiota* | *-* | 2 | 1.51 | 0.463 | 0.001 | 0.046 |
| Total | 249 | *Proteobacteria* | *-* | 3 | -1.50 | 0.562 | 0.008 | 0.142 |
| Total | 249 | *Bacteroidota* | *-* | 2 | 1.51 | 0.634 | 0.018 | 0.171 |
| Total | 249 | *Verrucomicrobiota* | *Akkermansia* | 2 | 1.59 | 0.463 | 0.001 | 0.042 |
| Total | 249 | *Proteobacteria* | *Burkholderia.Caballeronia.Paraburkholderia* | 2 | -0.86 | 0.294 | 0.004 | 0.086 |
| Total | 249 | Firmicutes | Streptococcus | 3 | 1.01 | 0.393 | 0.010 | 0.143 |
| Total | 249 | *Firmicutes* | *Staphylococcus* | 2 | -1.39 | 0.575 | 0.017 | 0.166 |
| Total | 249 | *Actinobacteriota* | *Rothia* | 2 | 1.42 | 0.525 | 0.007 | 0.116 |
| Total | 249 | *Proteobacteria* | *Burkholderia.Caballeronia.Paraburkholderia* | 3 | -0.73 | 0.311 | 0.020 | 0.184 |
| Total | 249 | *Bacteroidota* | *Bacteroides* | 2 | 1.40 | 0.624 | 0.025 | 0.180 |
| Preterm | 169 | *Verrucomicrobiota* | *-* | 2 | 1.75 | 0.585 | 0.003 | 0.080 |
| Preterm | 169 | *Proteobacteria* | *Burkholderia.Caballeronia.Paraburkholderia* | 2 | -0.97 | 0.334 | 0.004 | 0.144 |
| Preterm | 169 | *Verrucomicrobiota* | *Akkermansia* | 2 | 1.80 | 0.587 | 0.003 | 0.109 |
| Preterm | 169 | *Proteobacteria* | *Burkholderia.Caballeronia.Paraburkholderia* | 3 | -0.99 | 0.366 | 0.007 | 0.202 |
| Full-term | 80 | *Proteobacteria* | *-* | 3 | -3.19 | 1.052 | 0.003 | 0.142 |
| Full-term | 80 | *Firmicutes* | - | 3 | 1.04 | 0.415 | 0.015 | 0.249 |

Associations were analyzed using MaAsLin2. Neonates feeding mode covariate set: sex (male/female), postnatal age (days), birth weight, gestational age, antibiotic exposure (yes/no), delivery mode (vaginal/cesarean) and pediatric disease (respiratory/circulatory/ immune/others). N: Number of subjects. -: data unavailable, Value: 1 = BF group (reference group); 2 = FF group; 3 = MF. group, it reports the correlation of classification features. Coef: coefficient, Coef indicates the fold change in relative abundance (2^Coef) from the reference group to the comparison group specified in the ''value'' column. SE: standard error, MaAsLin2: microbiome multivariate associations with linear models.

**Figure S1. Flow diagram of participants selection.**

**Figure S2. Rarefaction and Species accumulation curves.** (A) Y-axis represents the number of ASVs to which the extracted sequence belongs and X-axis represents the number of extracted sequences. (B) Y-axis represents the number of ASVs detected and X-axis represents the sample size.

**Figure S3.** **Relative abundance of gut microbiota in BF, FF and MF groups of 265 neonates at the level of phylum and genus.** X-axis represents different groups and Y-axis represents the relative abundance of gut microbiota. BF, breast-fed group; FF, formula-fed group; MF, mixed-fed group.

**Figure S4.** **The relative abundance of gut microbiota in the BF, FF, and MF groups among 265 neonates.** (A) Differences in relative abundance at the order level. (B) Differences in relative abundance at the family level. *P < 0.05, **P < 0.01, ***P < 0.001, ****P < 0.0001. BF, breast-fed group; FF, formula-fed group; MF, mixed-fed group.

**Figure S5.** **Associations between feeding methods and gut microbiota were analyzed using RDA and MaAslin2 analysis in 265 neonates.** (A) Environmental explanation of changes in gut microbiota at the genus level by RDA analysis. The values on the axes represent the proportion of total variation in microbial abundance explained by the axes. Green arrows represent environmental factors, with the length of the arrows representing the strength of the impact of these environmental factors on microbial variation. The longer the arrow, the greater the impact of the environmental factor; conversely, the shorter the arrow, the lesser the impact. (B) Heatmap of multivariable associations identified by MaAsLin2. Red indicates significant positive correlation and blue indicates significant negative correlation, with deeper colors indicating stronger correlation. The reference groups were as follows: BF for feeding methods (feeding methods2: BF vs FF; feeding methods3: BF vs MF); no antibiotic exposure for antibiotic exposure (no vs yes); and vaginal delivery for delivery mode (vaginal delivery vs cesarean section).

**Figure S6.** **Relative abundance of gut microbiota in BF, FF and MF groups of premature and full-term neonates at the level of phylum (A and C) and genus (B and D).** X-axis represents different groups and Y-axis represents the relative abundance of gut microbiota. BF, breast-fed group; FF, formula-fed group; MF, mixed-fed group.

**Figure S7. The relative abundance of gut microbiota in the BF, FF, and MF groups among 177 preterm neonates.** (A) Differences in relative abundance at the order level. (B) Differences in relative abundance at the family level. *P < 0.05, **P < 0.01, ***P < 0.001, ****P < 0.0001. BF, breast-fed group; FF, formula-fed group; MF, mixed-fed group.

**Figure S8. Gut microbiota diversity and relative abundance in the BF, FF, and MF groups in** **88 full-term neonates.** (A–D) Comparison of the α-diversity indices among three groups. (E) PCoA among the three groups. (F-H) Differences in relative abundance at the phylum, order, and family levels. (I-K) Differences in relative abundance at the genus level. *P < 0.05, **P < 0.01, ***P < 0.001, ****P < 0.0001. BF, breast-fed group; FF, formula-fed group; MF, mixed-fed group.

**Figure S9. Difference analysis of metabolic pathways in 88 full-term neonates.** (A-C) Metabolic pathway analysis based on pairwise comparisons among the three groups. The color represents the significance level, while the point size reflects the number of genes. (D) Differences in lipoic acid metabolism between the BF and FF groups. (E) Differences in citrate cycle (TCA cycle) between the BF and FF groups. Red: enriched in FF group; Green: enriched in BF group.
